# Nicotinamide riboside kinases display redundancy in mediating nicotinamide mononucleotide and nicotinamide riboside metabolism in skeletal muscle cells

**DOI:** 10.1016/j.molmet.2017.05.011

**Published:** 2017-05-29

**Authors:** Rachel S. Fletcher, Joanna Ratajczak, Craig L. Doig, Lucy A. Oakey, Rebecca Callingham, Gabriella Da Silva Xavier, Antje Garten, Yasir S. Elhassan, Philip Redpath, Marie E. Migaud, Andrew Philp, Charles Brenner, Carles Canto, Gareth G. Lavery

**Affiliations:** 1 Institute of Metabolism and Systems Research, 2nd Floor IBR Tower, University of Birmingham, Birmingham, B15 2TT, UK; 2 Centre for Endocrinology, Diabetes and Metabolism, Birmingham Health Partners, Birmingham, B15 2TH, UK; 3 Nestlé Institute of Health Sciences (NIHS), Lausanne, CH-1015, Switzerland; 4 Ecole Polytechnique Fédérale de Lausanne, Switzerland; 5 Section of Cell Biology and Functional Genomics, Department of Medicine, Imperial College London, London, W12 0NN, UK; 6 Leipzig University, Hospital for Children and Adolescents, Center for Pediatric Research, Liebigstrasse 19-21, 04103, Leipzig, Germany; 7 Mitchell Cancer Institute, 1660 Springhill Avenue, Mobile, AL, 36604, USA; 8 School of Sport Exercise and Rehabilitation Sciences, University of Birmingham, Edgbaston, Birmingham, B15 2TT, UK; 9 Department of Biochemistry, Carver College of Medicine, University of Iowa, Iowa City, IA, 52242, USA

**Keywords:** Skeletal muscle, NAD^+^, Energy metabolism, Nicotinamide riboside

## Abstract

**Objective:**

Augmenting nicotinamide adenine dinucleotide (NAD^+^) availability may protect skeletal muscle from age-related metabolic decline. Dietary supplementation of NAD^+^ precursors nicotinamide mononucleotide (NMN) and nicotinamide riboside (NR) appear efficacious in elevating muscle NAD^+^. Here we sought to identify the pathways skeletal muscle cells utilize to synthesize NAD^+^ from NMN and NR and provide insight into mechanisms of muscle metabolic homeostasis.

**Methods:**

We exploited expression profiling of muscle NAD^+^ biosynthetic pathways, single and double nicotinamide riboside kinase 1/2 (NRK1/2) loss-of-function mice, and pharmacological inhibition of muscle NAD^+^ recycling to evaluate NMN and NR utilization.

**Results:**

Skeletal muscle cells primarily rely on nicotinamide phosphoribosyltransferase (NAMPT), NRK1, and NRK2 for salvage biosynthesis of NAD^+^. NAMPT inhibition depletes muscle NAD^+^ availability and can be rescued by NR and NMN as the preferred precursors for elevating muscle cell NAD^+^ in a pathway that depends on NRK1 and NRK2. Nrk2 knockout mice develop normally and show subtle alterations to their NAD+ metabolome and expression of related genes. NRK1, NRK2, and double KO myotubes revealed redundancy in the NRK dependent metabolism of NR to NAD^+^. Significantly, these models revealed that NMN supplementation is also dependent upon NRK activity to enhance NAD^+^ availability.

**Conclusions:**

These results identify skeletal muscle cells as requiring NAMPT to maintain NAD^+^ availability and reveal that NRK1 and 2 display overlapping function in salvage of exogenous NR and NMN to augment intracellular NAD^+^ availability.

## Highlights

• NRK1 and NRK2 are expressed in skeletal muscle and display redundancy in converting NR and NMN to NAD^+^.
• NRK1 and NRK2 are dispensable for maintaining basal skeletal muscle cell NAD^+^.
• Exogenous NMN salvage to NAD^+^ is NRK dependent.

## 1 Introduction

Nicotinamide adenine dinucleotide (NAD^+^) was first described as a vital cofactor in cellular redox reactions important to cellular energy metabolism [1], [2]. NAD^+^ also serves as a consumed substrate for enzymes such as sirtuins that post translationally modify proteins by deacetylation, yielding nicotinamide (NAM) and 2′-and 3-O-aceyl-ADP ribose in the process [3]. Sirtuins have been characterized as regulatory sensors that coordinate metabolic and transcriptional adaptations to cellular and tissue energy requirements [4], [5].

Skeletal muscle requires a high turnover of ATP to sustain contraction, facilitated by glycolysis and oxidative phosphorylation, which depend on the redox functions of NAD^+^ [6]. Because of the activity of NAD^+^ consuming enzymes, replenishment of NAD^+^ through biosynthesis and salvage pathways is vital [7], [8]. NAD^+^ can be synthesized de novo from tryptophan and by salvage of nicotinic acid (NA), a form of vitamin B3, via the Preiss-Handler pathway [9], [10]. Along with NA, nicotinamide (NAM) is also called vitamin B3 (collectively termed niacin) and as a nutrient or by recycling following NAD^+^ consumption, is metabolized by nicotinamide phosphoribosyltransferase (NAMPT) to nicotinamide mononucleotide (NMN), which is converted to NAD^+^ via NMN adenylyltransferases (NMNAT) [11]. A final route to NAD^+^ is the salvage and phosphorylation of the recently discovered form of vitamin B3 nicotinamide riboside (NR) to NMN, through the nicotinamide riboside kinase 1 and 2 (NRK1 and 2) pathway [2], [12], [13]. Despite mammalian cells demonstrating pathway diversity for maintaining NAD^+^ levels in various tissues, the relative contribution of these pathways to NAD^+^ biosynthesis in skeletal muscle remained unclear. The metabolic benefits of augmenting muscle NAD^+^ availability is being realized through the application of NAD^+^ precursor supplementation strategies. Historically, NA and NAM supplementation has been used to treat hypercholesterolemia and pellagra [14]. However, undesirable side effects in humans have limited their utility. NA causes flushing through activation of the GPR109A receptor in a large proportion of patients, leading to poor compliance [15]. Large doses of NAM are required to increase NAD^+^ leading to adverse effects and NAM mediated inhibition of sirtuin activity [16], [17]. The NA derivative Acipimox has shown the potential of using NAD^+^ precursors to improve and enhance mitochondrial function and ATP content of skeletal muscle in the context of diabetes [15].

NMN and NR have emerged as NAD^+^ precursors with the potential to circumvent the adverse side effects associated with high dose niacin and augment NAD^+^ synthesis and sirtuin activity [18]. NMN, being an intermediate of NAD^+^ biosynthesis, has been used to successfully ameliorate a number of pathological scenarios, including normalization of glucose tolerance in diet induced diabetes [19], and is able to restore mitochondrial function in aged muscle [20], [21]. Dietary supplementation of NR in mice can negate the metabolic consequences of high fat diet and increase oxidative performance [22], delay disease progression in mice with mitochondrial myopathy, inducing sirtuin dependent mitochondrial biogenesis and the mitochondrial unfolded protein response [23]. In addition, NR improves glycemic control and opposes development of diabetic neuropathy in mice and allows rats to resist development of chemotherapeutic neuropathy [24], [25]. Importantly, NR has been shown to safely elevate human NAD^+^ metabolism [26].

NAD^+^ precursor utilization pathways in muscle require further definition. Naturally available NR is phosphorylated to NMN by the NR kinases (NRKs encoded by *Nmrk1* and *2*) [12], [27], highly conserved enzymes, but little is known of their roles in skeletal muscle [12]. It is unclear whether NMN is truly available to muscle as an intermediate of NAD^+^ biosynthesis or is dependent on NRKs as a consequence of extracellular metabolism to NR prior to cellular uptake and incorporation into the cellular NAD^+^ pool [28]. Here we investigate NRK expression in skeletal muscle and define the influence of NRKs on NR and NMN metabolism to NAD^+^ in loss-of-function muscle cells derived from *Nmrk*1 and 2 knockout mice (NRK1KO, NRK2KO). We show that the NRKs have overlapping and redundant activity in muscle cells critical to the conversion of exogenous NR and NMN to NAD^+^.

## 2 Materials and methods

Unless otherwise specified all materials and reagents were acquired from Sigma–Aldrich, UK.

### 2.1 Animal care

Mice were group housed in a standard temperature (22 °C) and humidity-controlled environment with 12:12- hour light:dark cycle. Nesting material was provided and mice had ad libitum access to water and standard chow. Mice were sacrificed using schedule one cervical dislocation and tissues were immediately. All experiments were in groups of up to 6 and conducted within the UK Home office regulations.

### 2.2 Generation of NRK loss of function mice

NRK2KO mice were acquired from the Jackson Laboratory. The *Nmrk2* KO mutant allele was generated on a C57BL/6NTac background through the Knockout Mouse Phenotyping Program (KOMP2). A ZEN-UB1 Velocigene cassette (beta-galactosidase coding sequence from E. coli lacZ gene; polyadenylation signal; loxP site; promoter from the human ubiquitin C gene; neomycin phosphotransferase; polyadenylation signal; loxP site) was inserted through homologous recombination into the gene in place of all coding exons inhibiting transcription. Deletion of *Nmrk2* was validated by qPCR and immunoblotting.

NRK1KO mice generated on a C57BL/6NTac background have been previously described [28].

### 2.3 Exercise and fibre-typing

As a preliminary experiment, mice (n = 3) were acclimatized to the treadmill environment and exercised 3 times a week for 1 h at 0.25 M/Sec, at a 10° incline for 6 weeks.

Muscle sections were fiber typed using immunofluorescence following an established protocol [29], [30], [31]. Briefly, 10 μm sections were cut using a cryostat and mounted onto slides. Primary antibodies detecting different myosin heavy chain subunits (BA-F8 – MHC1 (1:50), BF-F3 – MHC IIb (1:100), SC-71 (1:600) – MHC IIa, 6H1 – MHC IIx (1:50)) (Developmental Studies Hybridoma Bank, University of Iowa) were added, followed by fluorescent secondary antibodies (IgG AF 647– Blue (1:500), IgM AF 555 – Red (1:500), IgG AF 488 – Green (1:500)). Sections were formalin fixed and mounted and then analyzed using a Zeiss Axio Observer inverted microscope (Carl Zeiss, Germany). Fibers were manually counted across the entire section using Image J (Fiji) software and recorded as positive for each fiber expressing the relevant visible color.

### 2.4 RNA extraction and qPCR

RNA was extracted from tissue and cells using TRI-reagent (Invitrogen). RNA quality was determined by visualization on a 1.5% agarose gel and quantified using a nanodrop. Reverse transcription was conducted using 500 ng RNA that was incubated with 250 μM random hexamers, 5.5 mM MgCl_2_, 500 μM dNTPs, 20 units RNase inhibitor 63 units multiscribe reverse transcriptase, and 1× reaction buffer. Reverse transcription was performed using a thermocycler set at the following conditions: 25 °C for 10 min and 37 °C for 120 min before the reaction was terminated by heating to 85 °C for 5 min qPCR was performed in a 384-well plate in single-plex format. Primers and probes were purchased as Assay on Demand (FAM) products (Applied Biosystems). Total reaction volumes used were 10 μl containing Taqman Universal PCR mix (Applied Biosystems). All Ct values were normalized to 18s rRNA (VIC) (Applied Biosystems). The real-time PCR reaction was performed under the following protocol: 95 °C for 10 min, then 40 cycles of 95 °C for 15 s, and 60 °C for 1 min using an ABI7500 system. Data were collected as Ct values and used to obtain deltaCt (dCt) values.

### 2.5 Western blotting

Protein lysates were extracted from tissues in RIPA buffer (50 mmol/l Tris pH 7.4, 1% NP40, 0.25% sodium deoxycholate, 150 mmol/l NaCl, 1 mmol/l EDTA) and protease/phosphatase inhibitor cocktail (Roche, Lewes, U.K.). Total protein concentration was quantified by Bio-Rad assay. Total proteins (25 μg) were resolved on a 12% SDS-PAGE gel and transferred onto a nitrocellulose membrane. Primary antibodies specific for NRK1/2 were generated and affinity purified by BioGenes (GmbH) Berlin, Germany and used at a 1:2000 dilution. Primary antibodies including NAMPT (Abcam, USA), β-Actin (Cell Signaling, USA, #12262) and α-Tubulin (Santa Cruz, USA, SC-5286) were all commercially available and used at a 1:1000 dilution. Secondary anti-mouse and anti-rabbit antibodies conjugated with HRP (Dako, Denmark) were added at a dilution of 1/10,000. Equal loading of protein content was verified using beta-actin and alpha-tubulin and bands visualized using ECL detection system (GE Healthcare, UK).

### 2.6 Preparation of tissue fractions

Muscle tissue was homogenized in sucrose buffer (0.25 M sucrose, 20 mM HEPES) and centrifuged at 1000 *g* for 10 min at 4 °C. The supernatant was transferred to a new tube and centrifuged at 12,000 *g* for 10 min at 4 °C. The mitochondrial fraction was pelleted and re-suspended in sucrose buffer and the supernatant was transferred to ultracentrifuge tubes and, following careful balancing, centrifuged at 100,000 *g* for 1 h at 4 °C. The supernatant containing the cytosolic fraction was transferred to a new tube and the pellet was re-suspended in MOPs buffer (100 mM KCl, 20 mM NaCl, 1 mM MgCl2, 20 mM MOPs) and washed 3 times by pelleting at 100,000 *g*. The microsomal pellet was re-suspended in MOPs buffer and snap frozen.

### 2.7 Primary muscle culture

Gastrocnemius tissue was excised from hind limbs of mice and digested in DMEM with 0.2% collagenase at 37 °C for 2 h. Digested tissue was washed in DMEM, and media was expelled vigorously onto the muscle to allow single myofibers to detach. Myofibers were then placed in pre-set matrigel (BD biosciences) coated wells in DMEM supplemented with 30% (v/v) FCS, 10% (v/v) HS, 1% (v/v) P/S, 2 mM l-glutamine, 1% (v/v) chick embryo extract (CEE) (Seralab, UK), and 0.1% of fibroblast growth factor (FGF) (Peprotech). Following satellite cell migration, media was replaced with proliferation media (DMEM with 10% (v/v) HS, 1% (v/v) P/S and 0.5% (v/v) CEE). Upon 70–80% confluence, media was replaced with Differentiation media (DMEM supplemented with 2% (v/v) HS, 0.5% (v/v) CE,E and 1% (v/v) P/S) and cells were differentiated for 6 days.

### 2.8 Cell treatments

Cells were treated in serum free media with 1 μM FK866 or DMSO as a vehicle control for 24, 48 or 72 h. Cells were supplemented with 0.5 mM NR (ChromaDex, USA), 0.5 mM NAR, 0.5 or 5 mM NAM and 0.5 mM NMN for 24 h. A concentration of 0.5 mM for cell treatments was used to provide a maximal effect on cellular NAD^+^ content following preliminary dose response experiments and previously published work in myotubes [22].

### 2.9 NAD^+^ measurements

#### 2.9.1 NAD^+^ by cycling assay

NAD^+^ was extracted from primary myotubes and quantified using EnzyChrom NAD/NADH Assay kit (BioAssay Systems) according to the manufacturer's instructions.

#### 2.9.2 NAD^+^ by HPLC

Total NAD was measured by reversed-phase HPLC using the Chromaster Purospher STAR RP-18 endcapped 3 μm Hibar RT 150-3 HPLC column (Merck). Frozen mouse muscle tissue (10 mg) was lysed in 100 μl 1 M perchloric acid. Samples were incubated on ice samples for 10 min, centrifuged and the supernatant was neutralized with 3 M potassium carbonate. After repeated centrifugation, samples were analyzed as previously described [32].

### 2.10 Targeted NAD^+^ metabolomics

20 mg of pulverised tissue samples were placed in Eppendorf tubes on dry ice. Metabolites were extracted in 0.2 ml of ice cold LCMS grade methanol and kept on ice before adding 300 μl of internal standard (diluted 1:300 in LC-MS grade water). Samples were briefly sonicated in an acetone water bath (at −4 °C), returned to ice, and then incubated at 85 °C for 5 min, shaking at 1050 rpm. Samples were cooled on ice for 5 min and centrifuged. Supernatant was dried using a speed vacuum. The dried extract was resuspended in 40 μl of either LC-MS grade water for acid extract or 10 mM ammonium acetate for alkaline extract and transferred to a Waters Polypropylene 0.3 ml plastic screw-top vial and analyzed by LC-MS/MS [33] using the ACQUITY UPLC H-class system.

### 2.11 Respirometry

C2C12 cells were differentiated into fully fused myotubes for 5 days. Respiratory output and mitochondrial stress tests were performed with C2C12 myotubes in XF-Assay media (Agilent Technologies) supplemented with 25 mM glucose, 0.5 mM sodium pyruvate, pH 7.4, maintained for 1 h at 37 °C in 0% CO_2_ prior to Seahorse extracellular flux analysis. Extracellular flux analysis was performed using the Seahorse XF analyser following manufacturer's instructions (Agilent Technologies).

### 2.12 Cell viability and apoptosis

C2C12 cells were seeded in a 96 well plate and differentiated for 5 days. Cell viability and apoptosis were assessed using the ApoLive-Glo™ multiplex assay (Promega) according to the manufacturer's instructions.

### 2.13 Statistical analysis

Unpaired Students *t*-test or ANOVA statistical comparisons were made using GraphPad Software Inc. Prism version 6. Data are presented as mean ± SEM with statistical significance determined as * = P < 0.05, ** = P < 0.01, *** = P < 0.001. Statistical analysis of real-time PCR data was determined using dCt values.

## 3 Results

### 3.1 NRK2 expression exhibits highly conserved skeletal muscle specificity

**Figure 1:**
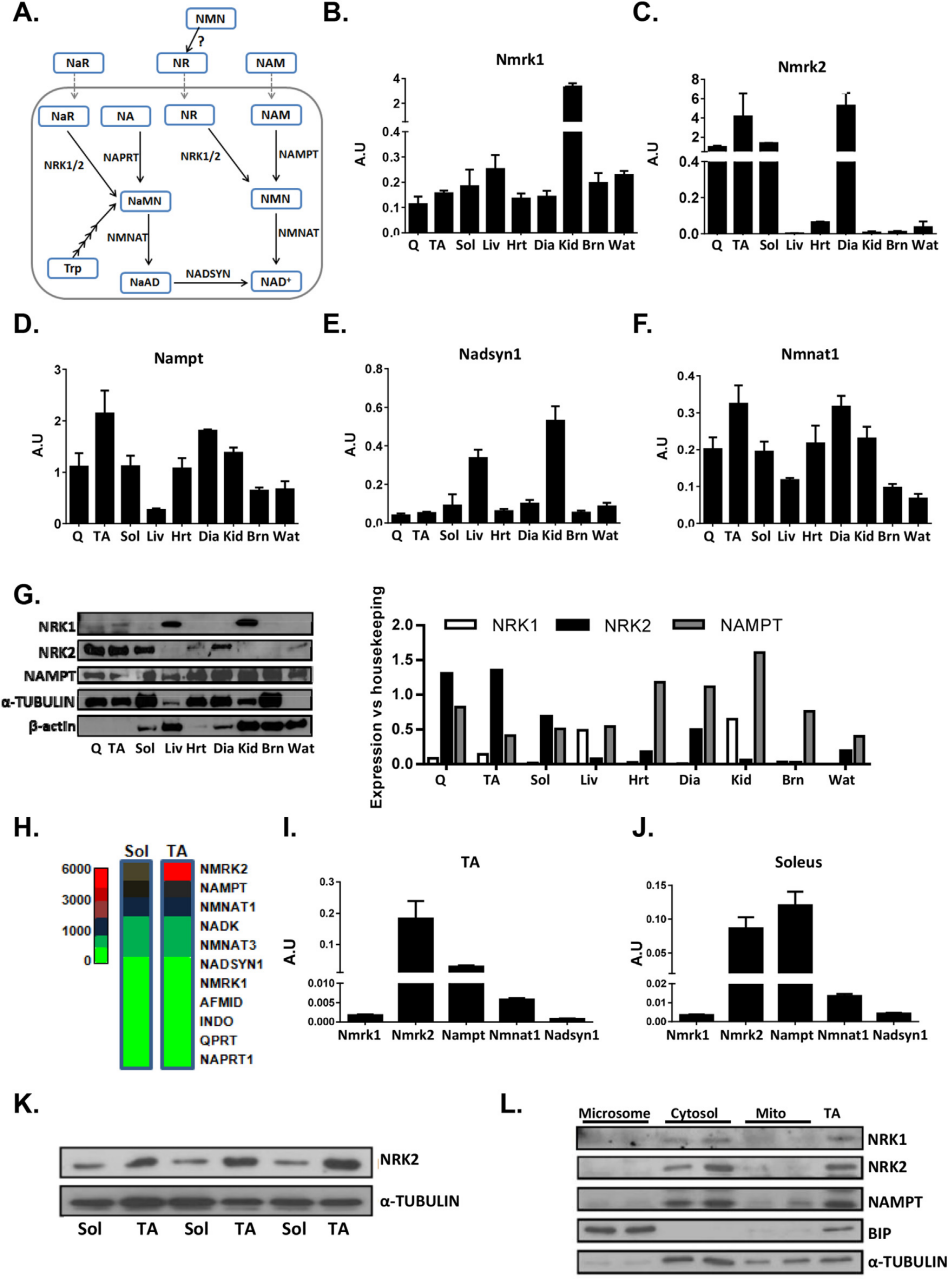
**NAD**^**+**^**biosynthesis pathways in skeletal muscle. (A)** An illustration of the known NAD+ biosynthesis pathways identified in mammalian cells with exogenous precursor supplementation strategies illustrated with dashed lines. NAD^+^ can be synthesized de novo from tryptophan (Trp) and following multiple enzymatic reactions is converted to nicotinic acid mononucleotide (NaMN) and then nicotinic acid dinucleotide (NaAD) by nicotinamide mononucleotide adenylyltransferase (NMNAT) activity, before the final conversion to NAD^+^ by NAD synthase (NADSYN). NAD+ can be salvaged from precursors nicotinic acid riboside (NaR) and nicotinic acid (NA) by nicotinamide riboside kinases (NRK1/2) and nicotinic acid phosphoribosyltransferase (NAPRT) respectively to NaMN and follow the same final steps. Finally, nicotinamide riboside (NR) and nicotinamide (NAM) are salvaged by NRKs and nicotinamide phosphoribosyltransferase (NAMPT) respectively to nicotinamide mononucleotide (NMN) and finally to NAD^+^ by NMNAT activity. Real-time PCR mRNA expression of *Nmrk1***(B)**, *Nmrk2***(C)**, *Nampt***(D)**, Nadsyn1 **(E),** and Nmnat1 **(F)** across metabolic tissues. **(G)** Protein expression of NRK1, NRK2, and NAMPT across metabolic tissues with corresponding densitometry compared to housekeeping protein (α-Tubulin for all tissues except liver and Wat which are compared to β-actin expression). **(H)** Microarray of NAD^+^ biosynthesis genes in tibialis anterior (TA) and soleus (Sol) skeletal muscle tissue (n = 3). Real-time PCR mRNA expression of rate limiting NAD biosynthesis genes in TA **(I)** and soleus **(J)** muscle (n = 4). **(K)** NRK2 protein expression in TA and soleus muscle tissue. **(L)** NRK1, NRK2, and NAMPT protein expression in cytosolic, mitochondrial and microsomal fractions of skeletal muscle. (Q = quadriceps, TA = tibialis anterior, Sol = soleus, Liv = liver, Hrt = heart, Dia = diaphragm, Kid = kidney, Brn = brain and Wat = white adipose tissue).

NAMPT and NADsyn1 act as rate limiting enzymes in the best characterized mammalian NAD^+^ biosynthesis pathways, with NADsyn1 the common final step in de novo synthesis and Preiss-Handler salvage pathways (Figure 1A) [34]. NRK 1 and 2 were more recently identified as NAD^+^ salvage enzymes involved in a novel NR dependent route to NAD^+^ [12] (Figure 1A). To better understand the roles the NRKs play in the context of the rate limiting enzymes NAMPT and NADsyn1, we examined their expression in a range of tissues to determine tissue specificity.

Using real-time PCR we show that *Nmrk1* is ubiquitously expressed, with particularly high expression in kidney, while *Nmrk2* exhibits high specificity to skeletal muscle (Figure 1B,C). *Nampt* is ubiquitously expressed across different tissue types as previously described (Figure 1D) [35], and *NADsyn1* shows greatest expression in liver and kidney tissue, consistent with previous data (Figure 1E) [36]. Nmnat1, common to all NAD^+^ biosynthetic pathways for conversion of NMN to NAD^+^, is ubiquitously expressed (Figure 1F). We went on to endorse these findings using Western blot analysis. While low levels of NRK1 are detectable in muscle, it was prominently detected in whole kidney and liver lysates (Figure 1G). For NRK2 we confirmed its muscle specificity. We also confirmed the ubiquitous expression of NAMPT (Figure 1G).

Using previously generated microarray data for soleus and tibialis anterior (TA) skeletal muscle [37], we further evaluated the relative expression of NAD^+^ biosynthesis and salvage genes. We identified *Nmrk2*, *Nampt*, and *Nmnat1* as the most abundantly expressed genes in soleus and TA muscle (Figure 1H). We used real-time PCR to confirm these findings and show that *Nmrk2* is the most predominantly expressed NAD^+^ biosynthesis gene in fast twitch tibialis anterior muscle compared to slow twitch fibre rich soleus muscle (Figure 1I,J). Western blots of soleus and tibialis anterior muscle lysates corroborated with the mRNA expression analysis, suggesting some muscle beds are enriched with NRK2 (Figure 1K). Finally, we addressed subcellular localization of the NRKs and NAMPT. Following fractionation of skeletal muscle tissue enriched into microsomes, cytosol, and mitochondria we show that NRK2, NRK1, and NAMPT are predominantly localized to cytosol (Figure 1L).

Although the role of NAMPT-mediated NAD^+^ biosynthesis has been characterized in skeletal muscle [38], [39], the function of NRK1 and NRK2 in particular, given its muscle restricted expression, and their relative contribution to NAD^+^ biosynthesis and homeostasis remained ill defined.

**Figure 2:**
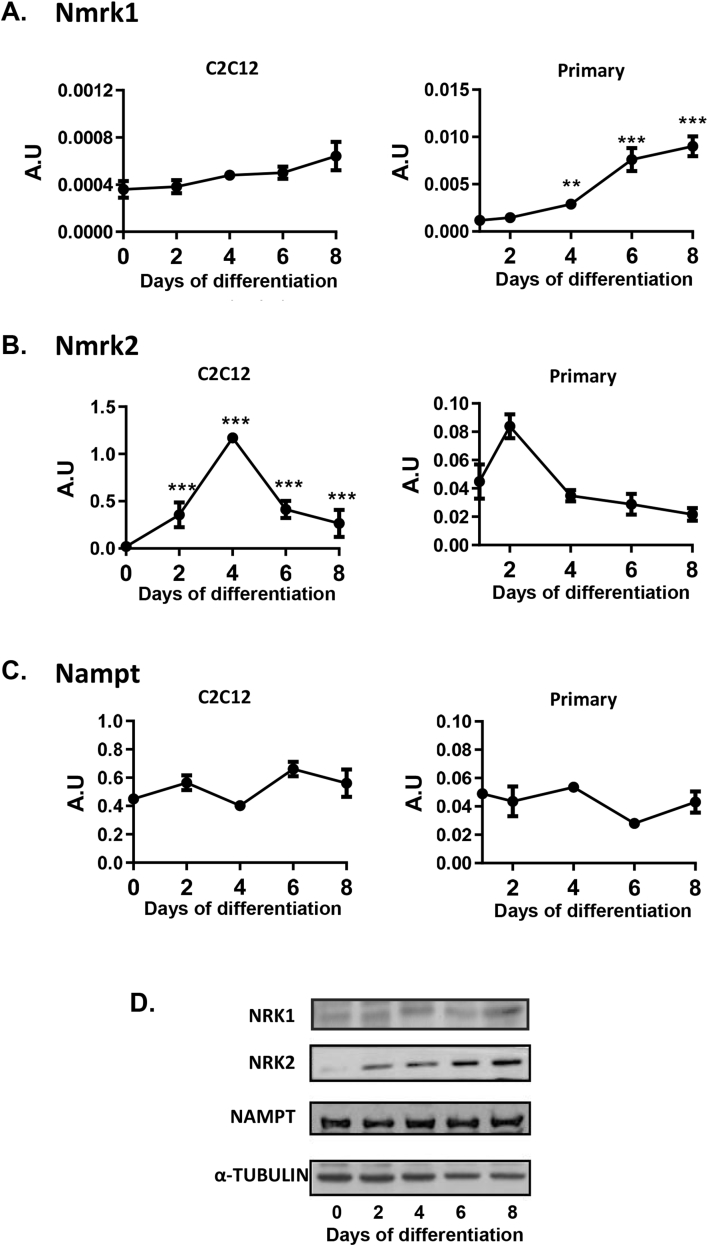
**Patterns of NAD**^**+**^**biosynthesis gene expression during skeletal muscle differentiation and muscle development**. 8 day differentiation time course of *Nmrk1***(A)**, *Nmrk2***(B)**, and *Nampt***(C)** mRNA expression in C2C12 and primary myotubes (n = 3–4). 8-day C2C12 myotube differentiation time course of NAD^+^ biosynthesis enzyme protein expression **(D)**.

To further examine the specific roles of the NRKs, we investigated mRNA and protein expression during C2C12 and primary muscle myotube differentiation. In C2C12 cultures, NRK1 demonstrated low level expression with respect to NRK2, rising to maximal expression at day 8 (Figure 2A,D). In contrast, NRK2 expression was upregulated during differentiation, peaking at day 4 around the time of myotube fusion (Figure 2B,D). NAMPT expression remained constant throughout differentiation (Figure 2C,D). We expanded this analysis to examine primary cultures of muscle cells derived from hind limbs of WT mice as a more physiologically relevant scenario. We used Myogenin (MyoG), Myogenic differentiation 1 (MyoD), and α-actin 1 (ACTA1) as markers of differentiation in our primary system, with all genes displaying anticipated profiles of expression for normal muscle cell differentiation (Sup. 1A). *Nmrk1*, *Nmrk2*, and *Nampt* showed similar patterns of differentiation as seen in C2C12s (Figure 2A–C). In addition, we showed that Nmnat1 is constitutively expressed during primary myotube differentiation (Sup. 1A). These data support the notion of a highly conserved and specific function for NRK2 in skeletal muscle.

### 3.2 NAD^+^ turnover in skeletal muscle is predominantly regulated by NAMPT salvage of NAM, yet NR proves a more valuable precursor for augmenting NAD^+^

As the NA Preiss-Handler pathway is mostly restricted to liver NAD^+^ biosynthesis and NA treatment is limited due to adverse effects, we aimed to establish the NAD^+^ precursors and the pathways that could be used to enhance NAD^+^ content in skeletal muscle [15], [34]. Metabolic enzyme activity profiling data suggest that the amidated routes to NAD^+^, mediated by the NAMPT and NRK salvage pathways, prevail as the main biosynthetic routes to NAD^+^ in muscle, which is corroborated by our tissue expression data and molecular analysis [34].

**Figure 3:**
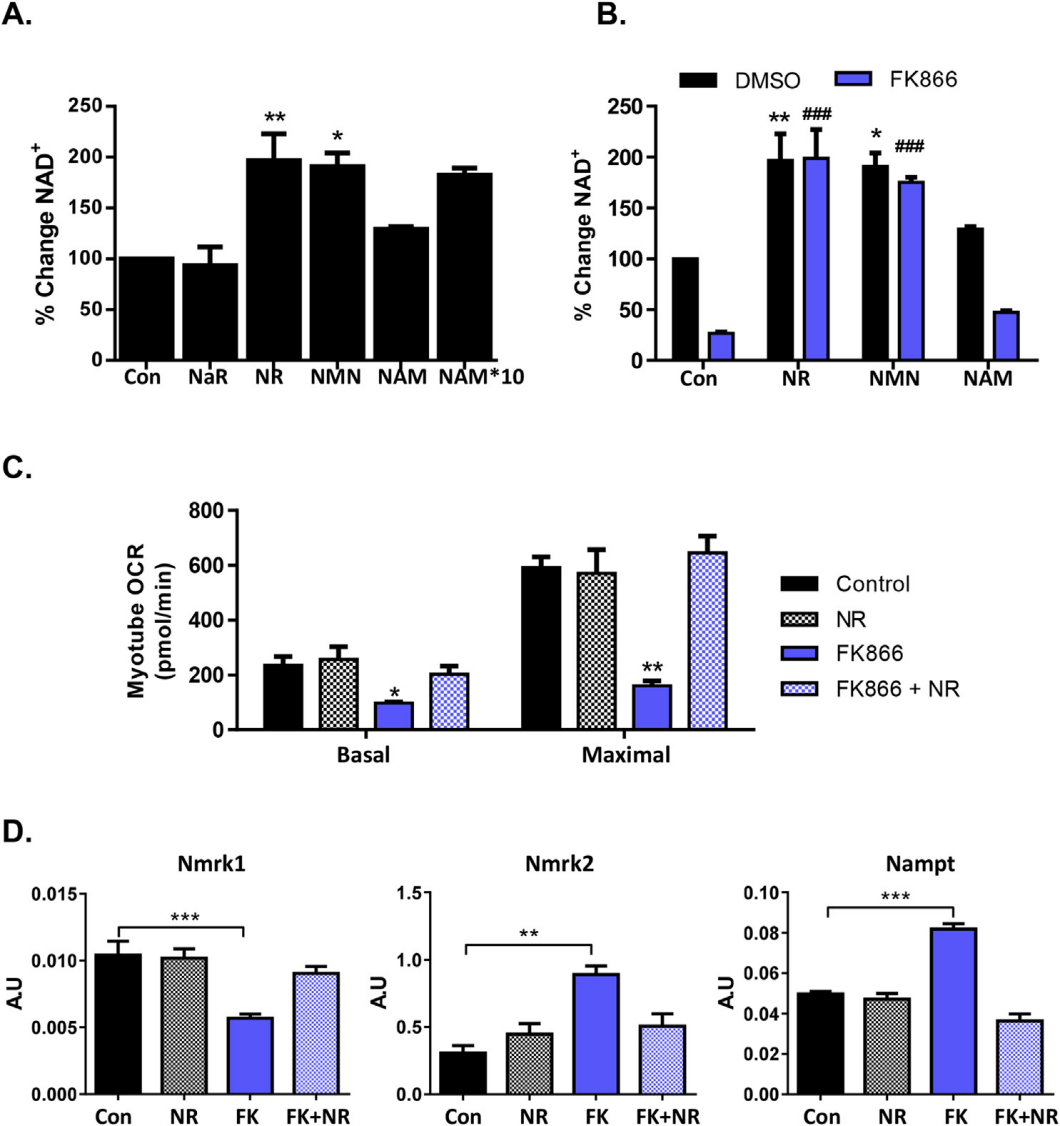
**Precursor availability and skeletal muscle NAD**^**+**^**biosynthesis. (A)** Primary myotube NAD^+^ levels following 24 h 0.5 mM NAD^+^ precursor supplementation (n = 4) determined using an NAD^+^ cycling assay. **(B)** Primary myotube NAD^+^ content following 24 h 0.5 mM NAD precursor supplementation with or without FK866 inhibition of NAMPT (* = significant from DMSO control and # = significant from FK866 treated control) (n = 4) determined using the NAD^+^ cycling assay. **(C)** Basal and maximal mitochondrial respiration following 24 h NR supplementation with or without FK866 inhibition (n = 3). **(D)** Transcriptional regulation of NAD^+^ biosynthesis genes, *Nmrk1*, *Nmrk2*, and *Nampt* following 24 h NR supplementation with or without FK866 inhibition of NAMPT (n = 3).

To further assess this, we supplemented primary-derived myotubes with NAD^+^ precursors that could be utilized by NRK or NAMPT pathways; NAR (non-amidated version of NR), NR, NMN, and NAM (Figure 1A) and quantified NAD^+^ using a cycling assay. NAR, like NR, is metabolized by NRKs to NAMN and then to NAAD by NMNATs [40]. However, NAAD requires final amidation to NAD^+^ via NAD synthetase [41]. We found that NAR was unable to augment the NAD^+^ pool in muscle cells, whereas both NR and NMN supplementation significantly increased NAD^+^ in myotubes by almost 2-fold. However equivalent concentrations of NAM did not significantly enhance NAD^+^, with 10-fold excess NAM required to increase NAD^+^ (Figure 3A). NAMPT is considered the rate limiting step for NAD^+^ salvage and synthesis in skeletal muscle [38] but has not been explored in detail in the context of NRKs. We examined precursor salvage and NAD^+^ levels in myotubes treated with the potent NAMPT inhibitor FK866 [42]. NAMPT is indeed essential for basal NAD^+^ homeostasis with NAD^+^ levels severely depleted (by more than 70%) following 24 h of inhibition. This depletion was completely reversed by NR and NMN supplementation, and both precursors were still able to boost NAD^+^ to the same extent as without FK866 treatment confirming that NR and NMN salvage is independent of NAMPT (Figure 3B).

To gain further insight into the activity of NR, we used LC-MS-based targeted quantitative metabolomics. Table 1 shows levels of key NAD^+^ metabolites in primary myotubes treated with FK866 and NR. As expected, NAD(H) levels significantly increase following NR supplementation, and, importantly, here we identify a large increase in cellular NR and NMN from control cells 24 h following cell treatments. Interestingly, metabolites of NAD^+^ consumption, NAM and ADP ribose (ADPr), were not altered by NR treatment but levels were reduced following FK866-induced NAD^+^ depletion, suggesting NAD^+^ availability is only rate limiting to NAD^+^ signaling below normal levels (Table 1). NA and other metabolites from the Preiss-Handler and de novo NAD^+^ biosynthesis pathways were undetected in primary myotubes.

**Table 1:** Targeted LC-MS NAD^+^ metabolome of primary myotubes treated with NR (0.5 mM) and/or FK866 (100 nM).

Metabolites (pmol/mg)	Control	NR	FK866	FK866 + NR
NAD(H)	790.2 ± 54.3	1968.1 ± 518.1*†	253.8 ± 25.7	1564.4 ± 207.6†
NADP(H)	90.5 ± 3.1	186.4 ± 62.1	55.1 ± 23.5	98.4 ± 20.1
NR	4.9 ± 0.6	73.6 ± 9.5*†	9.9 ± 2.0	112.1 ± 11.2*†
NAM	834.2 ± 29.8	1064.2 ± 172.4†	354.1 ± 43.3*	973.1 ± 75.8†
ADPr	535.3 ± 198.4	400.2 ± 54.1	326.6 ± 86	537.5 ± 104.3
NMN	ND	107.9 ± 26.5*†	3.8 ± 0.7	79.6 ± 12.1*†
Na	ND	ND	ND	ND
NaR	ND	1.7 ± 0.2*†	0.1 ± 0.1	1.9 ± 0.2*†
NaAD	ND	14.9 ± 5.6*†	ND	8.4 ± 2
NaMN	ND	ND	ND	ND

*Significant to control, †significant to FK866 treated, n = 3.

NAD^+^ is vital to mitochondrial respiration so we demonstrated the importance of NAMPT for maintaining NAD^+^ turnover by treating C2C12 myotubes with FK866 for 72 h. Inhibition of NAMPT significantly reduced basal and maximal respiration, which was fully rescued by treatment with NR for the final 24 h (Figure 3C). NR alone did not enhance mitochondrial respiration above untreated levels (Figure 3C). Concordantly, apoptosis was stimulated in C2C12 myotubes after 48–72 h of NAMPT inhibition. NR supplementation completely prevented this effect (Sup. 1B).

Changes in NAD^+^ availability and muscle cell energetics as a result of NAMPT inhibition are known to stimulate elevated rates of NAMPT gene transcription through AMPK activity; however, the effects on NRKs in this context are unknown [43], [44]. Using real-time PCR, we show that cellular mRNA expression of NAD^+^ salvage genes is tightly regulated by NAD^+^ availability. NAD^+^ depletion by FK866 resulted in upregulation of *Nmrk2* and *Nampt* and downregulation of *Nmrk1* (Figure 3D). Repletion of NAD^+^ by NR supplementation returned mRNA levels of all genes back to that of untreated control and NR treated cells (Figure 3D).

These data demonstrate that NAMPT is crucial for basal NAD^+^ biosynthesis. NR supplementation can completely rescue NAMPT inhibition through NRK-mediated NAD^+^ salvage. These data suggest that NRK activity is independently capable of maintaining cellular NAD^+^ and that NR availability may restrict NRK activity under basal conditions.

### 3.3 Loss of NRK2 function does not impair skeletal muscle mass or fiber type distribution in young mice

**Figure 4:**
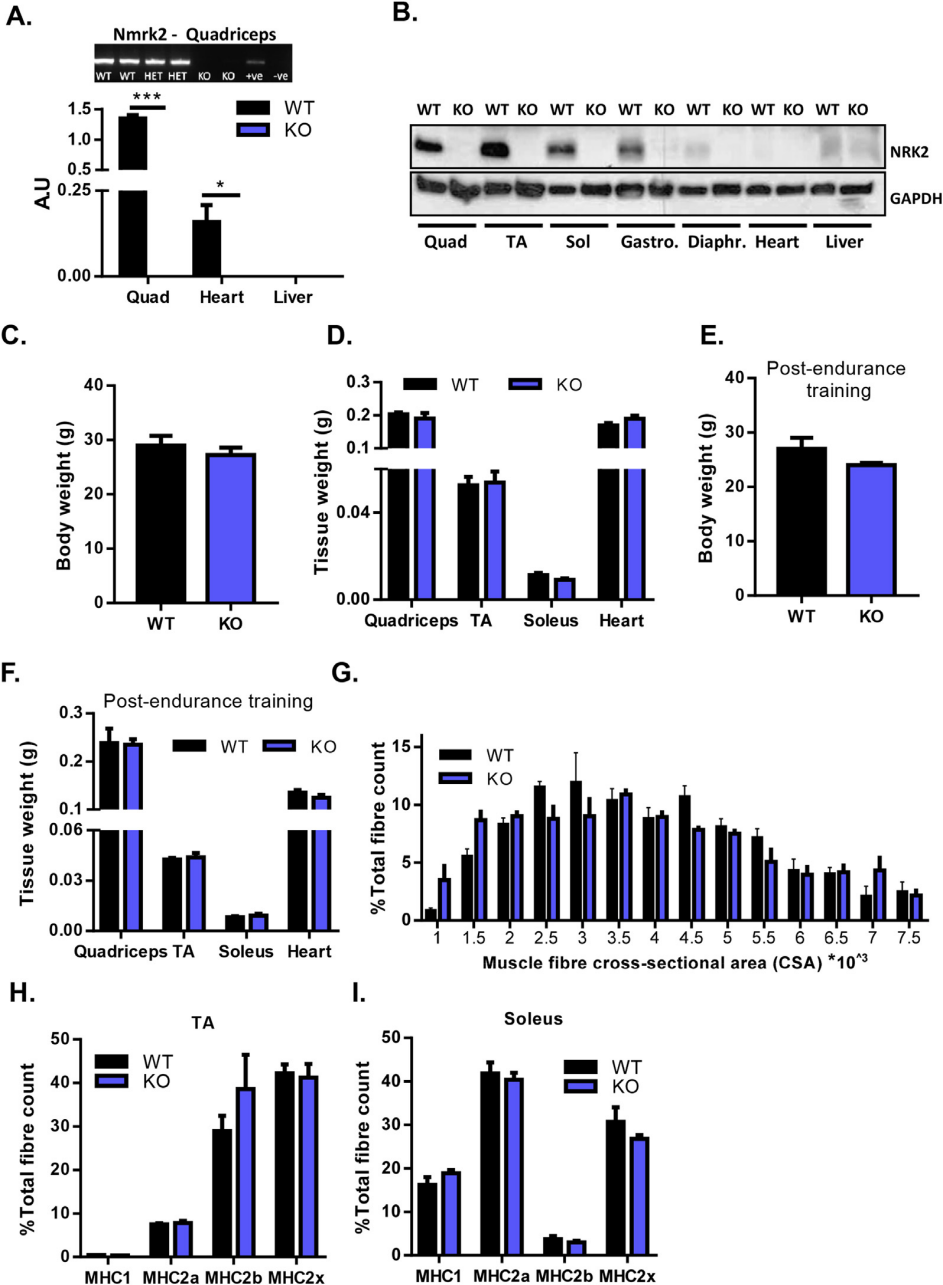
**Validation and characterization of NRK2KO mice. (A)** mRNA expression of *Nmrk2* in WT and NRK2KO quadriceps muscle, heart, and liver tissue. **(B)** Protein expression of NRK2 across metabolic tissues from WT and NRK2KO mice. **(C)** Whole body weight of 12 week old WT and NRK2KO mice. **(D)** Tissue weight of skeletal and cardiac muscle from 12 week old WT and NRK2KO mice. **(E)** Whole body weight of WT and NRK2KO mice following 6 weeks of endurance exercise training. **(F)** Tissue weight of skeletal and cardiac muscle from WT and NRK2KO mice following 6 weeks of endurance exercise training. **(G)** Muscle fiber cross-sectional area as a proportion of all fibers in WT and NRK2KO quadriceps. Fiber-type composition of TA **(H)** and soleus **(I)** skeletal muscle from WT and NRK2KO mice following 6 weeks of endurance exercise training. (All data n = 3–6).

To further elucidate the roles of the NRKs in skeletal muscle NAD^+^ salvage, we focused on the muscle-specific importance of NRK2 and have generated a model with constitutive ablation of the *Nmrk2* gene (NRK2KO). NRK2KO mice were born at anticipated Mendelian ratios and displayed no gross abnormalities. No *Nmrk2* mRNA or protein could be detected in any NRK2KO tissue examined, which included a range of skeletal and cardiac tissues (Figure 4A–B). 12–14 week old male and female mice were equivalent to WT control mice in terms of total body weight and lean mass for a range of muscle beds (Figure 4C–D). To induce metabolic stress and examine a role for NRK2 in skeletal muscle metabolic adaption, mice were subjected to an endurance exercise protocol. Again, total body weight and lean mass for a range of muscle beds were unchanged in NRK2KO mice (Figure 4E–F). Assessment of muscle fiber cross-sectional area showed no significant differences in proportions of fibre size in NRK2KO quadriceps compared to WT (Figure 4G). Similarly, muscle fiber type distribution assessed for myosin heavy chains (MHC) MHC1, MHC2a, MH2b, and MHCX in slow twitch rich soleus, and fast twitch rich TA muscle were normal and at expected proportions (Figure 4H–I).

### 3.4 NRK2 deficiency minimally impacts the skeletal muscle NAD^+^ metabolome

**Figure 5:**
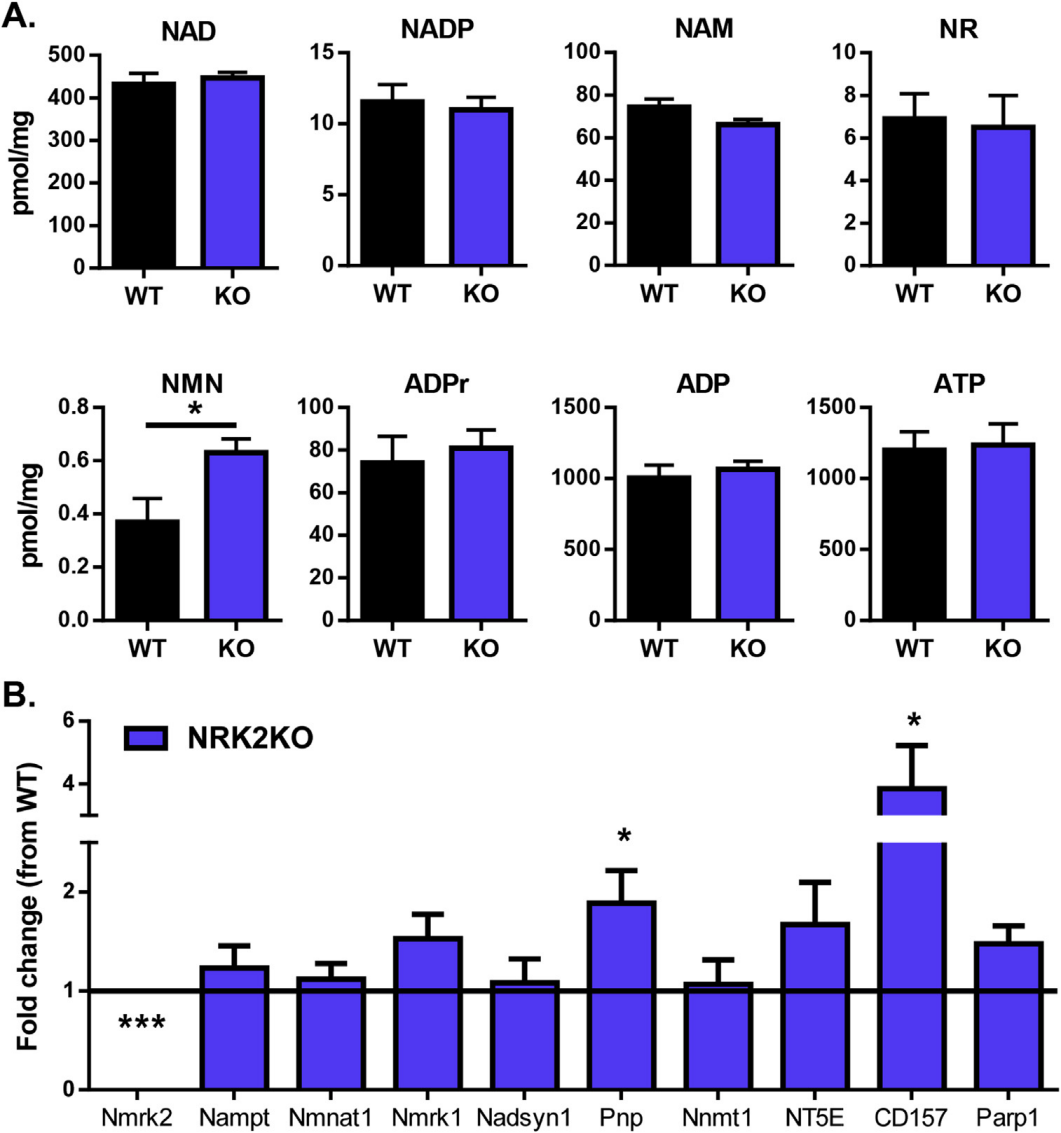
**NAD**^**+**^**availability and signaling in NRK2KO mice. (A)** LCMS-based targeted NAD^+^ metabolomics to quantify NAD^+^ and related metabolite levels in WT and NRK2KO skeletal muscle tissue (n = 6). **(B)** Fold change in mRNA expression of NAD^+^ related signaling genes in NRK2KO skeletal muscle compared to WT (at Y axis = 1) (n = 4).

Recent evidence has shown that NR can elevate muscle NAD^+^ when administered to mice or cultured cells [22]. To ascertain the effect of NRK2 deficiency on muscle levels of NAD^+^ and related metabolites we used LC-MS-based targeted quantitative NAD^+^ metabolomics [33]. This method allows quantification of NAD^+^ as well as associated metabolites to determine the wider effect loss of NRK2 has on NAD^+^ metabolism. Surprisingly, NAD^+^ levels in quadriceps tissue from NRK2KO mice were shown not to be deficient compared to WT control tissue (Figure 5A). The NAD^+^ precursors NR and NAM were unchanged, but a significant increase in NMN was detected (Figure 5C). Furthermore, no differences in NADP, ATP, ADP, or the NAD^+^ consumption product ADPr were detected (Figure 5A). To confirm the NAD^+^ data generated by LC-MS, NAD^+^ levels were independently measured using a colorimetric cycling assay and by HPLC [32], which confirmed that there were no significant alterations to tissue NAD^+^ and NADH levels (Sup. 1C–D).

Finally, we examined the expression of key NAD^+^ salvage and biosynthetic genes in response to loss of *Nmrk2*. We did not detect any adaptive changes in *Nmrk1* or *Nampt* but did detect increased expression of Pnp (2-fold) and CD157 (4-fold) (Figure 5B). Purine Nucleoside Phosphorylase (PNP) is involved in purine metabolism and has activity to convert NR to NAM [45]. CD157 has NAD^+^ nucleotidase activity liberating cADP-ribose and NAM [46]. These adaptive responses in gene expression, to compensate for loss of NRK2 in living mice, may have more long-term consequences for NAD^+^ salvage and require further assessment.

### 3.5 NRKs are essential for exogenous NR and NMN utilization to NAD^+^ in cultured muscle cells

To better understand the interactions and contributions of muscle NRK 1, NRK2, and NAMPT to NR and NMN precursor salvage we employed a primary muscle culture system to derive myotubes with combinatorial single, double, and triple NRK1/2 and NAMPT loss of function.

These experiments were conducted using NRK1 and 2KO mice as reported by Ratajczak et al. [28]. Firstly, due to the highly muscle-specific nature of NRK2, we assessed expression of key muscle differentiation markers and NAD^+^ signaling genes over myotube differentiation in all the NRKKO models and found that expression was not altered, confirming that the NRKs are not essential for myotube differentiation (Sup. 2A,B).

**Figure 6:**
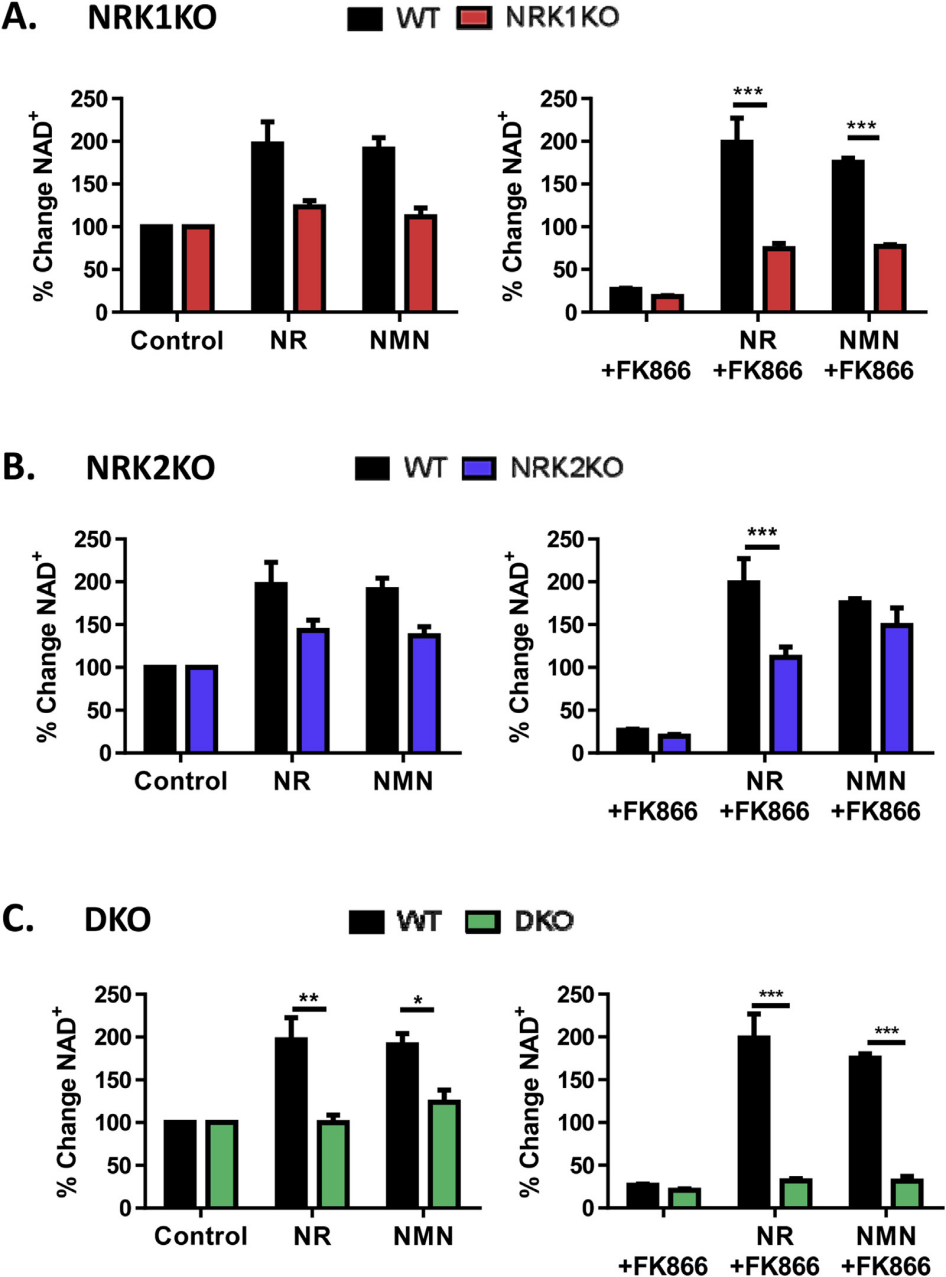
**Manipulations to skeletal muscle NAD**^**+**^**biosynthesis pathways. (A)** NRK1KO, **(B)** NRK2KO, and **(C)** NRK double KO (DKO) primary myotubes supplemented with 0.5 mM NAD^+^ precursors for 24 h (left) and corresponding primary myotubes supplemented with 0.5 mM NAD^+^ precursors for 24 h following 1 μM FK866 mediated NAMPT inhibition (right). For clarity, all data is presented as percent change in NAD^+^ compared to DMSO only control (100%) (all groups n = 4 and significance determined between WT and corresponding KO from actual NAD^+^ content by two-way ANOVA using Bonferroni's post-test).

Basal NAD^+^ levels in NRK single or double KO (DKO) cells were comparable to those of WT cells (Sup. 2C). Similarly, but to a lesser extent than in the WT myotubes, single NRK1KO and NRK2KO myotubes were still able to enhance NAD^+^ levels following NR supplementation, more so in NRK2KO cells compared to NRK1KO cells (43.25% increase in NAD+ versus 23.5%), suggesting functional redundancy of the NRKs to generate NAD^+^ from available NR. However, NR was unable to increase NAD^+^ in double KO cells with less than a 1% change from untreated (Figure 6A–C, left).

In hepatocytes, extracellular NMN is converted to NR extracellularly such that both NR and NMN require NRK1 activity to convert these compounds to intracellular NMN and NAD^+^ [28]. In unstressed NAD^+^ replete cells NMN supplementation could not augment NAD^+^ levels in NRK1KO, and to a lesser degree NRK2KO, such that double KO cells are effectively unresponsive to NMN (Figure 6A–C, left).

We then examined the ability of NR and NMN to rescue cells depleted of NAD^+^ following 24 h NAMPT inhibition (Figure 6A–C, right). NAMPT inhibition substantially depleted cellular NAD^+^ content in WT and all the NRK loss of function cell cultures (Figure 6A–C, right). NR and NMN were able to recover, but not boost, NAD^+^ levels following FK866 inhibition in NRK2KO and, to a lesser extent, NRK1KO myotubes. However, neither NR nor NMN supplementation was able to recover NAD^+^ depletion in DKO myotubes, again clearly indicating the NRK dependency of NMN conversion to NAD^+^ (Figure 6A–C, right). In all instances, 10-fold excess NAM (5 mM) is required to overcome NAMPT inhibition (Sup. 2D).

These data show that while NAMPT is the primary pathway for maintaining NAD^+^ turnover in skeletal muscle, the NRKs are essential for utilizing exogenous NR and NMN to enhance cellular NAD^+^.

## 4 Discussion

Mounting evidence supports the notion that enhancing tissue NAD^+^ availability has beneficial effects on metabolic health in the context of metabolic disease and physiological decline [22], [23], [47], [48], [49]. The leading method to accomplish NAD^+^ augmentation is through dietary supplementation with NR and NMN. Although they have a similar capacity to raise NAD^+^ and promote metabolic benefit, the tissue-specific routes of their metabolism remain obscure. Skeletal muscle exhibits a decline in NAD^+^ in a number of physiological, metabolic, and genetic scenarios, all associated with defects in muscle physiology and mitochondrial function [7], [50], [51]. Here we have explored NAD^+^ salvage and biosynthesis in skeletal muscle and identified NRKs as critical to the availability of both NR and NMN to NAD^+^ in skeletal muscle cells.

NAD^+^ biosynthesis in skeletal muscle appears restricted to two main salvage pathways involving the rate-limiting enzymes NAMPT, NRKs, and NMNAT. While basal NAMPT and, to a degree, NRK1 display ubiquitous expression, NRK2 displays a muscle restricted pattern at mRNA and protein level. De novo NAD^+^ biosynthesis and the Preiss-Handler NA salvage pathway play a minimal role in skeletal muscle with limited or undetectable levels of expression of rate limiting enzymes and metabolites. This supports previous metabolic enzyme activity profiling data that found enzyme activity of NAD synthase – the final rate limiting enzyme for both de novo biosynthesis and NA salvage – to be undetectable in skeletal muscle [34]. We endorse this by showing that unlike NR, the acid version NaR, which is metabolized by the NRKs and NMNATs – but also requires NAD synthase activity for final conversion to NAD^+^ [40], [41] – does not appear able to act as an exogenous NAD^+^ precursor in skeletal muscle.

Depletion of NAMPT activity in muscle leads to a severe reduction (85%) in muscle NAD^+^ availability; yet, at least for young mice, no gross NAD^+^ relatable phenotype was observed [38]. However, with advancing age (7 months), this chronic reduction in NAD^+^ manifests with impaired mitochondrial function, loss of muscle fiber integrity, strength, and performance [38]. Although NAMPT is critical to basal NAD^+^ biosynthesis, exogenous NAM appears to be a poor precursor for NAD^+^ boosting in muscle compared to NR and NMN due to poor conversion to NAD^+^ and its inhibitory activity towards sirtuins [52]. We found that NR supplementation could significantly boost NAD^+^ in the context of reduced NAD^+^ levels following NAMPT inhibition and rescue concurrent defects in mitochondrial energy metabolism. Despite NR rescuing NAD^+^ depletion and some of the effects of metabolic challenge *in vivo* [22], our data suggest that increasing NAD^+^ availability in skeletal muscle by NR supplementation does not enhance oxidative metabolism of ‘healthy’ NAD^+^ replete muscle cells. Muscle-specific NAMPT overexpressing mice showed that a 50% increase in NAD^+^ could not stimulate mitochondrial biogenesis or enhance mitochondrial metabolism in young skeletal muscle [53]. Our results show that when NAD^+^ is enhanced through NR supplementation, NAM and ADPr levels are unchanged, whereas there is a decrease following NAD^+^ depletion (Table 1). As NAM and ADPr are metabolites of NAD^+^ signaling (products of SIRT based NAD^+^ consumption) [54], this indicates that although a reduction of NAD^+^ can limit NAD^+^ signaling and ultimately hinder mitochondrial metabolism, basal NAD^+^ content alone is not limiting to NAD^+^ signaling, at least in healthy skeletal muscle.

Though NRK2 appears to be restricted to muscle, loss-of-function had no effect on basic parameters of muscle physiology, perhaps reflecting the unstressed nature of young muscle in this study, and in line with the work of Frederick et al. [38]. Again, this also suggests that NAMPT is more than sufficient to match NAD^+^ recycling to its metabolic clearance. Measuring the quadriceps tissue NAD^+^ related metabolome was unremarkable other than for a 50% increase in NMN. Similarly, NAMPT and NRK1 were unaffected at the level of gene expression and may suggest that no overt stress response was initiated. However, we did note elevation in the expression of *Pnp* and *CD157*. PNP can convert NR to NAM and CD157 converts extracellular NAD+ to ADPr and NAM [45], [46]. While this does not manifest as raised NAM level in muscle, it may reflect raised NMN that serves to balance NAD^+^ availability suggesting a minor adaptation to the loss of NRK2.

We originally postulated that NRK2 would be more important than NRK1 in skeletal muscle for NR salvage due to its predominant expression. However, our *in vivo* data and work by Ratajczak et al. showing that NRK1 controls NR and NMN metabolism in mammalian cells [28] led us to examine more closely the dependency of NR and NMN on each NRK. Using combinatorial muscle cultures of single and double NRK1/2 KO cells we confirmed that NR is exclusively metabolized by the NRK enzymes in skeletal muscle with no change to cellular NAD^+^ levels in DKO primary myotubes following NR supplementation, which was strikingly evident in NAD^+^ depleted cells. Both NRK1 and NRK2 single KO cells demonstrate a level of redundancy in their ability to respond to NR, though clearly being submaximal compared to control cells.

NMN is metabolized to NAD^+^ downstream of NRKs and NAMPT, yet exogenous NMN supplementation was unable to be metabolized to NAD^+^ in DKO cells, again most strikingly in the context of severe NAD^+^ depletion. While there is a level of redundancy in the single KO cells, the data implicate NRK1 as being more critical for regulating NMN entry into the cell. Recent findings by Ratajczak et al. have shown that NRK1 regulates exogenous NMN salvage in liver tissue and cells. They identified a requirement for NMN to be metabolized extracellularly to NR before hepatic uptake, then re-phosphorylated by NRK1 back to NMN [28]. Similar data also have highlighted that human cells can metabolize NMN to NR intracellularly utilizing cytosolic 5′-nucleotidases [40]. The data presented here in primary WT and loss-of-function myotubes corroborate these findings and reveal the NRKs critical role for both NR and NMN conversion to NAD^+^.

Positive health effects of enhancing NAD^+^ availability have been demonstrated in muscle [55]. Acipimox (NA analogue) treatment improved aspects of mitochondrial physiology in type 2 diabetic muscle. However, the effects on NAD^+^ availability were small and the mechanisms unclear, and the therapeutic potential of acipimox limited due to a range of adverse effects including insulin resistance and GPR109A receptor activation leading to flushing and therefore poor compliance [15]. The inhibition of PARPs, sparing and elevating NAD^+^, leading to enhanced mitochondrial function and biogenesis through activation of SIRT1-PGC-1α axis has been demonstrated as being effective in muscle [56], [57], [58].

However, supplementation with NR and NMN to boost cellular NAD^+^ pools in a range of scenarios appears to be the most promising strategy [19], [20], [22], [48], [51], [59], [60], [61], [62], [63]. NR and NMN based NAD^+^ repletion in skeletal muscle can revitalize stem cells and augment physiological and mitochondrial function in aged mice [49]. Furthermore, NAD^+^ can also act to regulate protein–protein interactions that influence DNA repair through control of PARP activity, and NMN or NR repletion of NAD^+^ in ageing could support efficient DNA repair capacity while maintaining the positive effects of sirtuin activation [64].

While NR is naturally available in the human diet, with appreciable levels measured in milk, the main source of NR, NMN, NAM, and NA is the NAD(P) (H) pool available through the digestion of whole, unprocessed food [27]. Oral NR administration to humans has recently demonstrated effectiveness in boosting circulating and white blood cell NAD^+^ levels, with further work required to determine the importance for muscle NAD^+^ repletion [26].

These data establish that muscle has a level of redundancy in its ability to metabolize NR and therefore NMN. Despite NRK2 being restricted to muscle and being highly expressed, basal NAD^+^ turnover does not seemingly require NRK activity. However, NRK1 and to a smaller degree NRK2 are gate-keepers of NR and NMN salvage. Importantly, NAD^+^ depletion and energetic stress can lead to augmented NRK2 expression in a range of tissues such as neurons, cardiac, and skeletal muscle [65], [66], [67]. Thus, beyond basal NAD^+^ turnover, regulated NRK2 activity may serve an additional role in cellular metabolism involving NR phosphorylation, potentially more critical to stress adaptation when there is need to enhance NR/NMN metabolism to defend metabolic integrity.

## Author contributions

GGL and CC conceived and designed the study. RSF, JR, CLD, and LAO performed the experiments. RC, GSX, AG, and YE contributed to experiments. PR, MM, and CB contributed reagents, technology and critical evaluation of the manuscript. RSF, AP, and GGL wrote and CB edited the manuscript.

## Appendix A Supplementary data

Sup 1 **(A)** mRNA expression of Nmnat1, common to all NAD biosynthesis pathways, and muscle differentiation marker genes; MyoG, MyoD, αActin over an 8 day primary muscle cell differentiation time course (n = 4). **(B)** Cell viability (left axis, black lines) and apoptosis (right axis, blue lines) following 72 h FK866 treatment with (solid line) or without (dashed line) 24 h NR supplementation (n = 2). **(C)** NAD^+^ cycling assay determination of skeletal muscle NAD^+^, NADH and NAD/NADH ratio in WT and NRK2KO mice (n = 4). **(D)** Total NAD levels in WT and NRK2KO skeletal muscle tissue determined by HPLC. **(E)** Representative images showing MHC expression. Cross section (i–iv) incubated with BA-F8 and BF-F3 (CK1a) along with corresponding secondary antibodies, showing MHC I (blue) and IIB (red) expressing fibres. Cross section (v–viii) incubated with SC-71 and BF-F3 (CK1b) along with corresponding secondary antibodies, showing MHC IIA (green) and IIB (red) expressing fibres. Cross section (ix–xii) incubated with SC-71 and 6H1 (CK2) along with corresponding secondary antibodies, showing MHC IIA (green) and IIX (purple) expressing fibres. Sup 1

Sup 2 mRNA expression of markers of skeletal muscle differentiation **(A)** and skeletal muscle NAD^+^ biosynthesis genes **(B)** in WT, NRK1KO, NRK2KO, and DKO primary myotubes over an 8 day differentiation time course. **(C)** Basal NAD^+^ levels in WT, NRK1KO, NRK2KO, and DKO primary myotubes measured using an enzyme cycling assay. **(D)** Percent change in NAD^+^ of NRK double KO primary myotubes supplemented with 0.5 mM and 5 mM (*10) NAM for 24 h following 1 μM FK866 mediated NAMPT inhibition compared to DMSO only control (100%). (All data n = 4–5). Sup 2
