# Supplementary material for: Nicotinamide riboside kinases display redundancy in mediating nicotinamide mononucleotide and nicotinamide riboside metabolism in skeletal muscle cells
Source: Mol Metab. 2017 May 29;6(8):819–32. doi: 10.1016/j.molmet.2017.05.011 (PMC5518663; doi:10.1016/j.molmet.2017.05.011)
Supplement: Sup 1 — (A) mRNA expression of Nmnat1, common to all NAD biosynthesis pathways, and muscle differentiation marker genes; MyoG, MyoD, αActin over an 8 day primary muscle cell differentiation time course (n = 4). (B) Cell viability (left axis, black lines) and apoptosis (right axis, blue lines) following 72 h FK866 treatment with (solid line) or without (dashed line) 24 h NR supplementation (n = 2). (C) NAD+ cycling assay determination of skeletal muscle NAD+, NADH and NAD/NADH ratio in WT and NRK2KO mice (n = 4). (D) Total NAD levels in WT and NRK2KO skeletal muscle tissue determined by HPLC. (E) Representative images showing MHC expression. Cross section (i–iv) incubated with BA-F8 and BF-F3 (CK1a) along with corresponding secondary antibodies, showing MHC I (blue) and IIB (red) expressing fibres. Cross section (v–viii) incubated with SC-71 and BF-F3 (CK1b) along with corresponding secondary antibodies, showing MHC IIA (green) and IIB (red) expressing fibres. Cross section (ix–xii) incubated with SC-71 and 6H1 (CK2) along with corresponding secondary antibodies, showing MHC IIA (green) and IIX (purple) expressing fibres. [file mmc1.pptx]

## Slide 1
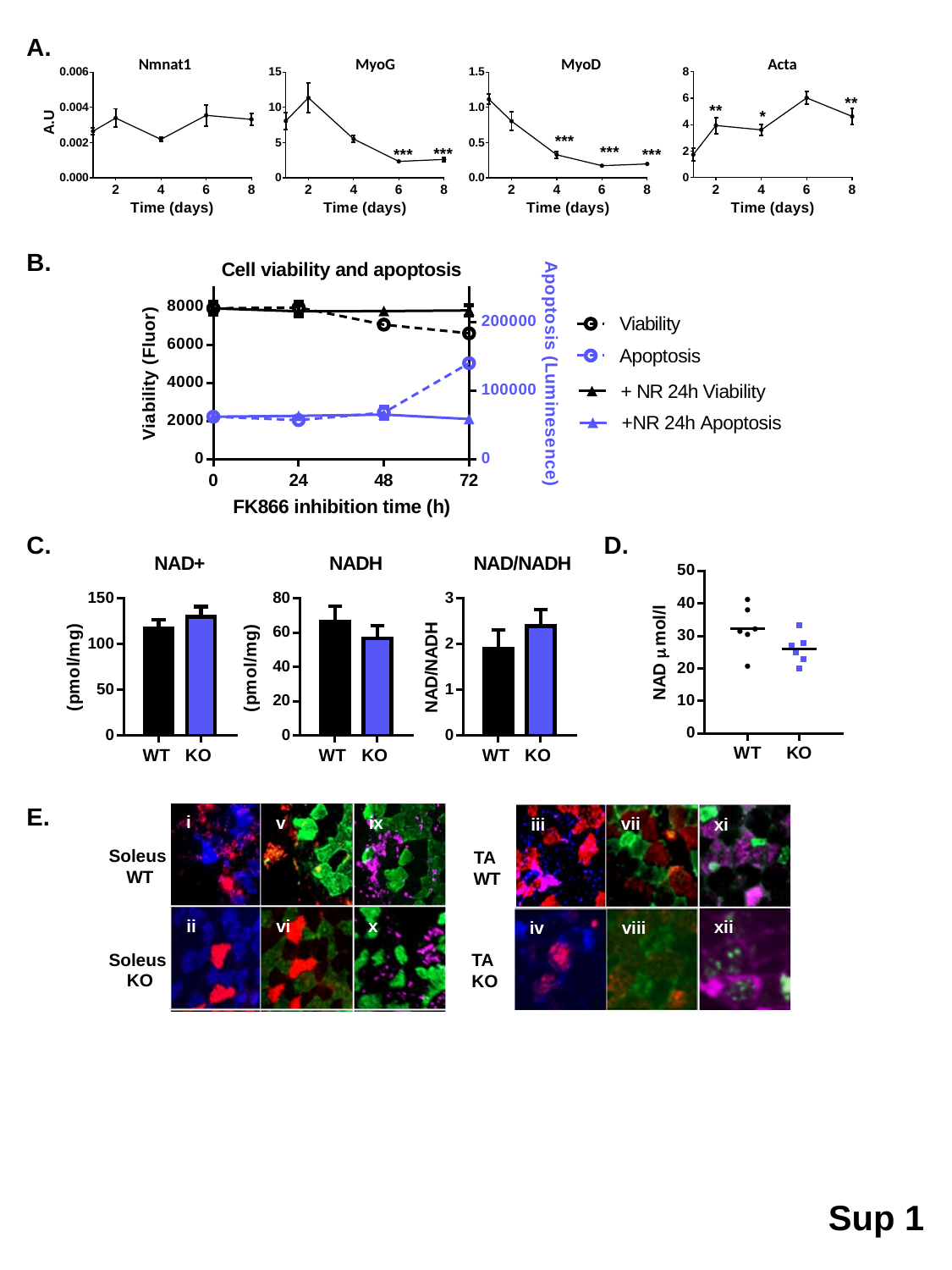

A.
Nmnat1
MyoG
MyoD
Acta
B.
C.
D.
E.
i
v
ix
vii
iii
xi
Soleus
WT
TA
 WT
ii
vi
x
xii
iv
viii
Soleus
KO
TA
KO
Sup 1
