# Supplementary figures and images for: Nicotinamide riboside kinases display redundancy in mediating nicotinamide mononucleotide and nicotinamide riboside metabolism in skeletal muscle cells
Source: Mol Metab. 2017 May 29;6(8):819–32. doi: 10.1016/j.molmet.2017.05.011 (PMC5518663; doi:10.1016/j.molmet.2017.05.011)

## Slide 1
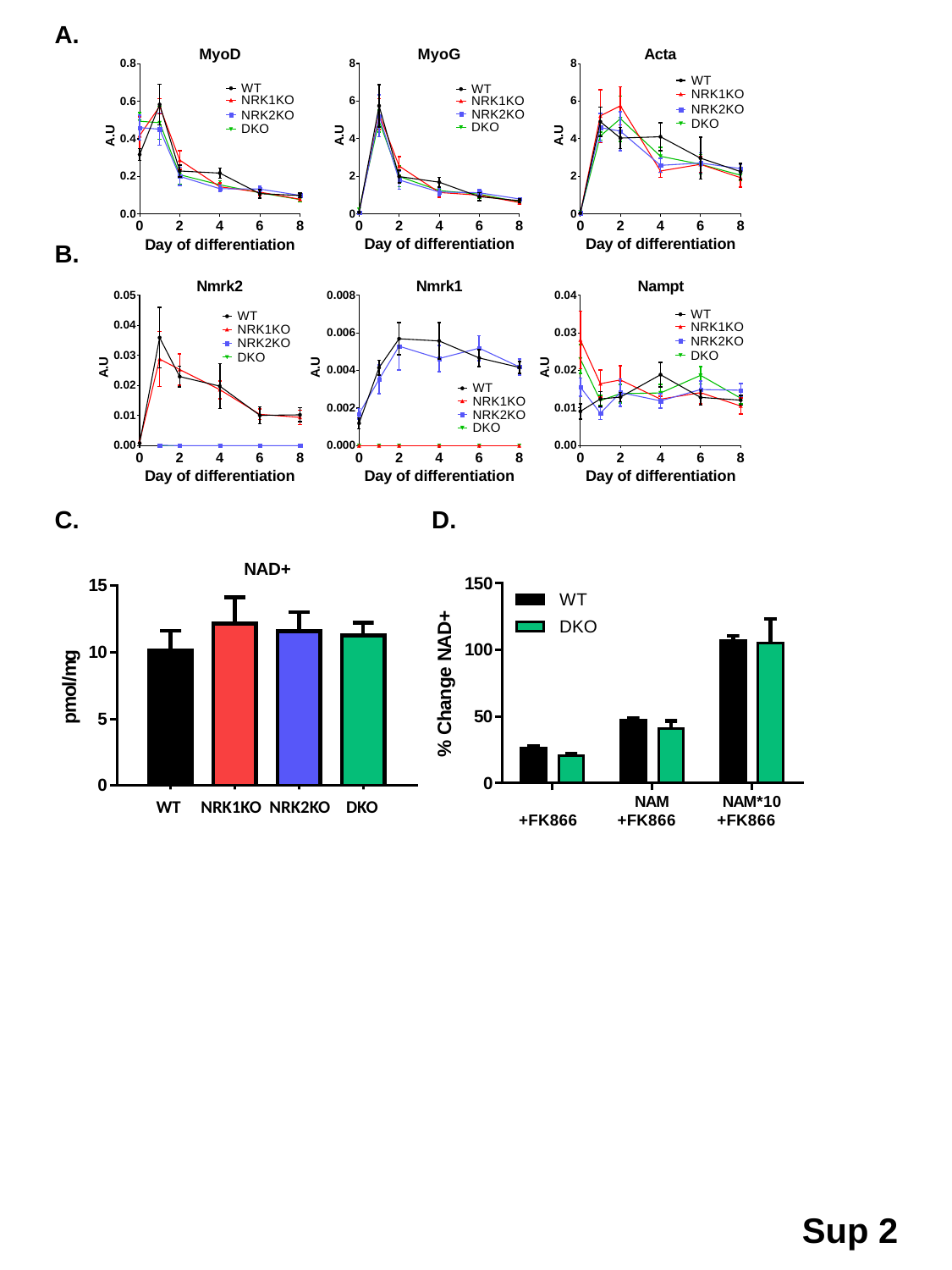

A.
B.
C.
D.
WT NRK1KO NRK2KO DKO
Sup 2

Supplement: Sup 2 — mRNA expression of markers of skeletal muscle differentiation (A) and skeletal muscle NAD+ biosynthesis genes (B) in WT, NRK1KO, NRK2KO, and DKO primary myotubes over an 8 day differentiation time course. (C) Basal NAD+ levels in WT, NRK1KO, NRK2KO, and DKO primary myotubes measured using an enzyme cycling assay. (D) Percent change in NAD+ of NRK double KO primary myotubes supplemented with 0.5 mM and 5 mM (*10) NAM for 24 h following 1 μM FK866 mediated NAMPT inhibition compared to DMSO only control (100%). (All data n = 4–5). [file mmc2.pptx]
